# A mouse is not a rat is not a man: species-specific metabolic responses to sepsis - a nail in the coffin of murine models for critical care research?

**DOI:** 10.1186/2197-425X-1-7

**Published:** 2013-10-29

**Authors:** Peter Radermacher, Philippe Haouzi

**Affiliations:** Sektion Anästhesiologische Pathophysiologie und Verfahrensentwicklung, Klink für Anästhesiologie, Universitätsklinikum, Helmholtzstrasse 8-1, D-89081 Ulm, Germany; Division of Pulmonary and Critical Care Medicine, Penn State University College of Medicine, Penn State Hershey Medical Center, Hershey, PA 17033 USA

“All models are wrong, but some are useful.”

-Box GEP, Draper NR (1987) Empirical model-building and response surface, Wiley

Sepsis and consequent multi-organ failure are the leading causes of mortality in critically ill patients. Numerous therapeutic strategies, which yielded promising results in preclinical studies, have failed to show any efficacy in clinical trials. Historically, most of our (patho)physiological understanding of cardiovascular regulation in health and disease has been established in larger mammals, e.g., dog, pig, and sheep. However, a very large body of the literature collected over recent decades originates from investigations in rodents. Despite their small size, which makes surgery difficult and limits repetitive blood sampling, murine models have been widely used, not because they are more faithful to adult human pathophysiology than larger mammals but because they are inexpensive, easy to handle and care for, and with availability of gene knockout and overexpression strains. Investigators have performed studies without necessarily having a good appreciation of some fundamental differences in the physiology of rodents. These have been long established by comparative physiologists, yet have never been taught in any medical school. One can query the logic of inferring information on mechanisms involved in septic shock using species capable of decreasing their high resting metabolic rate both rapidly and massively, and with the associated circulatory and respiratory responses.

Mouse models of acute inflammatory disorders have recently been questioned by Seok et al. [1] who found that “genomic responses to different acute inflammatory stresses are highly similar in humans”, whereas “these responses are not reproduced in current mouse models”. In this context, Zolfaghari et al. [2] compared the metabolic responses to polymicrobial sepsis in rats and mice. Their main findings were that mice presented with a progressive drop in whole body O_2_ uptake and a concurrent fall in body temperature, which was only partially restored by external warming. This marked metabolic depression coincided with pronounced impairment of left heart systolic contractility. In sharp contrast, only severely ill rats showed a comparably decreased cardiac output, and the metabolic depression was only present during the late premortem period.

How can we explain these findings by Zolfaghari et al. [2]? The authors have the merit of raising an issue of crucial importance for critical care research, i.e., whether or not murine models can yield sufficient information to enable a good design of clinical studies. In particular, they must be commended for having achieved not only an identical 72-h mortality but also comparable 24-h clinical severity scores. This is by no means trivial in the two species investigated: body weight differs by an order of magnitude, and mice present with a twofold to threefold higher metabolic expenditure on a per gram basis. Interestingly, albeit the final outcome was identical, the time course of the clinical development of sepsis differed: the clinical severity score was already higher in mice with “severe” sepsis at 6 h post inoculation of feces, whereas in rats, this difference was only apparent at 24 h. One might argue that the resuscitation technique may have assumed importance in this context: rats were infused with continuous i.v. fluids via a jugular venous catheter inserted prior to the experiment, while mice received intermittent s.c. bolus administration. Moreover, even sham-operated mice showed moderate hyperlactatemia, hyperglycemia, and hyperchloremia, and, in particular, marked metabolic acidosis. The latter is a common finding in mouse models reported by other authors [3, 4], even when balanced electrolyte solutions rather than saline were used as maintenance fluids [5, 6]. This prompted some authors to systemically administer bicarbonate infusions [7].

The authors' observation of markedly reduced energy expenditure and consecutive hypothermia in mice is not new and is most likely due to the fundamentally different metabolic response in this species rather than to any of the abovementioned phenomena: in response to tissue hypoxia, mice reduce their energy expenditure by decreasing “nonshivering thermogenesis”. Nonshivering thermogenesis [8] as a result of the high activity of mitochondrial uncoupling proteins [9] represents a very large proportion of overall O_2_ consumption which can therefore be depressed very rapidly without affecting ATP production. Such a regulation of metabolism represents a unique protective adaptation, observable in many species including human babies. Reduced VO_2_ can be observed during hemorrhagic shock or severe hypoxia in rats [10], but this response is less pronounced in adult individuals [11, 12]. In other words, even “small animal model species are not created equal” [13]. Hence, whenever interventions target or affect cellular energy metabolism [14, 15], results obtained in mouse models may be misleading. In keeping with their thermal and metabolic regulation, the physiology of an adult mouse is much closer to that of a premature newborn baby than an adult rat!

Consequently, should we abandon mouse models for critical care research and replace them with rats? Undoubtedly, rats are easier to handle than mice, and they have a 10 to 20 times higher blood volume. However, in rats, the response to injury may also markedly differ from that of humans, in particular with respect to mediators that are referred to assume crucial importance during sepsis. For example, rats present with blood concentrations of nitric oxide (NO) metabolites that are 10 to 20 times higher than those in humans. Moreover, they are particularly resistant to oxidative stress, a common phenomenon during sepsis, due to their high tissue activity of antioxidant enzymes [16, 17]. Indeed, there is an abundant literature on promising rat studies of antioxidants or NO inhibitors, which have never translated into clinical practice. However, this problem is inherent to the use of other “nonhuman” models; mice, for instance, also show different activity for inducible NO synthase (iNOS)-related NO release [18, 19]. As a consequence, one might consider only using large animal species, e.g., swine, dogs, or ewes. However, in addition to cost, labor, and specific ethical concerns of the lay public, there is no ideal large animal species either which fits all needs: The use of ruminants may be questioned when gastrointestinal function is to be investigated due to their different anatomy. Despite their general similarity with humans, swine can be a problem when they are used in targeting lung mechanics and perfusion due to a lack of collateral alveolar ventilation and a marked pulmonary hypertensive response.

Finally, due to their experimental design covering several days, Zolfaghari et al. [2] could not address the question whether it is necessary - and if so, to what extent - to integrate standard intensive care procedures into an experimental design, ultimately to guarantee clinical relevance for critical care research. The rationale for this discussion is self-evident and was highlighted by the late Prof. Daniel Traber more than a decade ago, “Would you…accept data on a septic patient who was not resuscitated…, …who did not even have blood pressures and heart rates monitored?” [20]. This point is essential since although murine models are very different from humans due to their own evolutionary specificities, when it comes to shock or its treatment, the persistence of fundamental responses shared by all mammals must be acknowledged. For instance, striking similarities exist between all species as well as through the different ages (from premature babies to elderly patients) as far as the principles of treatment of septic shock (antibiotics, volume replacement, etc.…) are concerned. Although the time course of the clinical development of sepsis differed between rats and mice, in the study of Zolfaghari et al. [2], the final outcome was identical in both species. Whenever standard ICU strategies are used [21, 22], the similarities between models become even more obvious, regardless of differences in physiological regulation, level of inflammation, temperature, and VO_2_ or NO regulation. For instance, as demonstrated by Hollenberg [22], the mortality of mice 48 h after cecal ligation and puncture was close to 100% without resuscitation, whereas fluids and antibiotic therapy resulted in 45% survival, a value close to the clinical setting. For obvious technical reasons, the smaller the species studied, the more challenging the intensive care-type measurements during an experiment are. Consequently, this problem is much more pronounced in murine than in rat models. The use (or lack) of mechanical ventilation or hemodynamic monitoring is a prominent example. The miniaturization of equipment will certainly allow many limitations to be overcome, but will of course not solve fundamental species-specific differences. Although it would be interesting to develop methods to reduce basal metabolic rate in humans based on the response seen a mouse model, relevant qualitative as well as semiquantitative information on the benefits of therapeutic strategies could still be obtained using small mammals. Similar to the vast majority of researchers, Zolfaghari et al. [2] used young, male, and otherwise healthy animals. These certainly do not represent the majority of ICU patients who are often elderly with chronic preexisting comorbidities. Age, gender, and comorbidities influence outcomes not only in patients but also in experimental models of sepsis [23–32].

Hence, as a bottom line conclusion, Zolfaghari et al. [2] highlighted an important issue of translational research in critical care medicine, i.e., that data on the physiological response to septic shock in a mouse or a rat model can only be understood in keeping with the frame of reference of the animal’s physiology. Such data cannot be directly extrapolated to septic patients. The hope is that by acknowledging and understanding these differences, the resources involved with murine models will not be wasted and that rodents could be continued to be used, with a rational frame of reference based on objective elements of comparative pathophysiology - rather than physiology - a discipline which is still in its very early age and which remains to be developed. Only then could mouse models take their real place in critical care research along with large animal investigations and *in vitro* (i.e., reductionist) approaches before commencement of any clinical trial.
